# Canopy structure, dry matter intake and performance of Holstein × Gyr cows (*Bos taurus taurus* L. × *Bos taurus indicus* L.) under rotational stocking in *Urochloa* spp. pastures

**DOI:** 10.1007/s11250-026-05169-5

**Published:** 2026-06-22

**Authors:** Natalia Avila Soares, Carlos Augusto de Miranda Gomide, Patrícia do Rosário Rodrigues, Domingos Sávio Campos Paciullo, Mirton José Frota Morenz, Angelo Herbet Moreira Arcanjo, Fernando Antônio de Souza, Fernanda de Kássia Gomes, Pedro Drummond Rodrigues, Ângela Maria Quintão Lana

**Affiliations:** 1 Department of Animal Science, Federal University of Minas Gerais, Av. Pres. Antônio Carlos, 6627, Belo Horizonte, 31270-901 MG Brazil; 2 Brazilian Agricultural Research Corporation (Embrapa Dairy), Av. Eugênio do Nascimento, 610, Juiz de Fora, 36038-330 MG Brazil; 3 Minas Gerais Agricultural Research Corporation (EPAMIG Oeste), Getúlio Vargas Experimental Station, Rua Afonso Rato, Uberaba, 1301, 38060-040 MG Brazil; 4 Minas Gerais Agricultural Research Corporation (EPAMIG Centro Oeste), Santa Rita Experimental Station, MG-424 Highway, km 64, Prudente de Morais, 35738-000 MG Brazil

**Keywords:** Dry matter intake, Herbage production, Leaf:stem ratio, Milk composition, Milk yield, Volumetric density

## Abstract

Pasture-based dairy systems require well-adapted forage cultivars and appropriate grazing management to sustain milk production while maintaining favorable sward structure. The objective of this study was to evaluate the performance of Holstein × Gyr crossbred cows and the productive and structural characteristics of Paiaguás grass pasture (*Urochloa brizantha* cv. BRS Paiaguás) and Ipyporã grass pasture (*Urochloa brizantha* × *Urochloa ruziziensis* cv. BRS Ipyporã), under rotational stocking during two rainy season. For the agronomic variables, a completely randomized design was adopted in a split-plot design: two grasses and three experimental phases, with twelve paddocks evaluated per phase. The arrangement for milk yield and milk composition was the complete switchback trial, and for dry matter intake, it was the cross-over. Paiaguás grass showed greater herbage mass, accumulation and herbage accumulation rate. Ipyporã grass showed a greater leaf: stem ratio (1.54 vs. 1.02) and greater volumetric density (127.45 vs. 108.15 kg DM cm ha^− 1^). Milk yield did not differ between cultivars or experimental phases with an average value of 107.65 L ha^− 1^ day^− 1^). Stocking rate was higher in Paiaguás grass pasture compared with Ipyporã grass (7.71 vs. 6.90 AU ha^− 1^). Dry matter intake differed between cultivars only in phase 2, when it was higher for Ipyporã grass compared with Paiaguás grass (2.21 vs. 1.97% of body weight, respectively). Both cultivars supported a great milk yield when managed with 41 cm pre-grazing height for cv. BRS Ipyporã and 63 cm for cv. BRS Paiaguás, and 50% defoliation.

## Introduction

Pasture-based dairy cow production systems are used worldwide, with different levels of intensification (Neal and Roche 2019; Boddey et al. 2020; Wilkinson et al. 2020; Oliveira et al. 2026). The use of these systems is associated with lower production costs, greater sustainability, higher product quality, and improved animal welfare (Hanrahan et al. 2018; Merino et al. 2020; Kogima et al. 2022). The low productivity is partly a consequence of intense monoculture, poor adaptation of the herbage to the growing environment, and inadequate management practices (Euclides et al. 2010; Souza et al. 2025). Under rotational grazing management, the adoption of a rest period based on reaching approximately 95% light interception by the herbage canopy (Parsons and Penning 1988; Da Silva and Nascimento Jr 2007), combined with 50% pasture reduction (Sbrissia et al. 2018), results in high net herbage production and low restriction to dry matter intake (DMI) by grazing animals.

Canopy structure is a key interface between herbage growth and animal response in rotationally stocked tropical pastures. Structural traits such as canopy height, leaf: stem ratio, dead material, and the arrangement of morphological components influence herbage harvesting and, consequently, DMI and milk production (Rezende et al. 2021). In pasture-based dairy systems with crossbred Holstein × Gyr cows, structural and morphological characteristics of the herbage canopy were more closely associated with milk yield per hectare than forage chemical composition alone (Rezende et al. 2021). Similarly, defoliation management based on canopy development regulates tissue turnover, leaf utilization, herbage-use efficiency, and stocking density in grazed swards (Gastal and Lemaire 2015).

The search for diversification and increased pasture productivity has led research to develop grass and legume cultivars with superior characteristics (Valle et al. 2009). Two recently released cultivars of great importance are *Urochloa brizantha* cv. BRS Paiaguás (syn. *Brachiaria brizantha* cv. BRS Paiaguás), which shows great persistence during the dry season, and *Urochloa* hibrida [*U. brizantha* x *U. ruziziensis* (syn. *Brachiaria* híbrida)] cv. BRS Ipyporã, the first *Urochloa* hybrid released by Embrapa, and standing out for its tolerance to pasture spittlebugs, the main insect pests of tropical pastures (Valle et al. 2013, 2017; Echeverria et al. 2016; Euclides et al. 2016, 2018).

Although BRS Paiaguás and BRS Ipyporã were developed for tropical grazing systems, information on their use with dairy cows remains limited, particularly when canopy structure, DMI, and animal performance are evaluated simultaneously. During pasture establishment in the Cerrado biome, tropical herbage cultivars differ in morphogenetic and structural traits, with Paiaguás and Ipyporã presenting contrasting establishment dynamics (Gurgel et al. 2022). Therefore, cultivar-specific information is essential for selecting appropriate herbage cultivars and for supporting annual planning and growth management in pasture-based dairy systems (Santos and Fonseca 2016). Addressing these gaps may refine grazing-management recommendations and improve sustainability and land-use efficiency in tropical environments (Macdonald et al. 2017).

The present study hypothesizes that morphological and nutritional differences between the cultivars result in variations in herbage production and milk productivity. Therefore, the objective of this study was to evaluate milk yield and DMI of Girolando cows, as well as the structural characteristics of Paiaguás grass and Ipyporã grass pastures under rotational stocking, managed at 93–95% of light interception.

## Materials and methods

### Experimental area

**Fig. 1:**
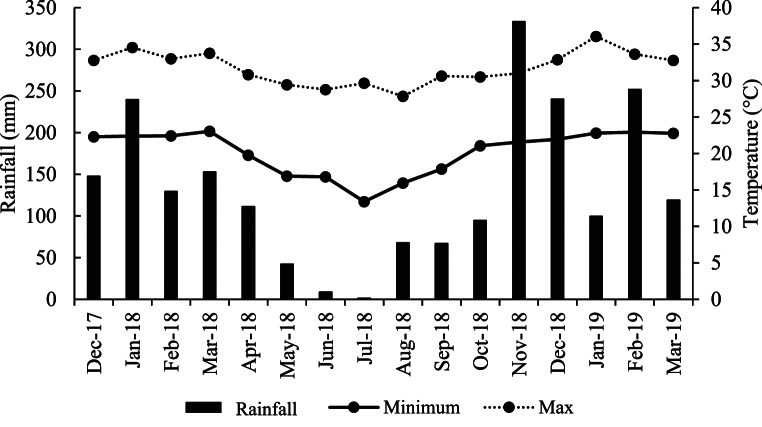
Precipitation, maximum and minimum temperature throughout the experimental period, (Data collected from Inmet, Station: Coronel Pacheco - A557)

The experiment was conducted in the Mata Atlântica biome at the José Henrique Bruschi Experimental farm of Brazilian Agricultural Research Corporation – Dairy Cattle (21°33’22’’S and 43°06’15’’W, altitude 410 m), from December 2017 to June 2019. The region climate in classified as *Cwa*, humid subtropical, following the classification proposed by Köeppen-Geiger (Alvares et al. 2013). Climate data were collected from the meteorological station of the National Institute of Meteorology (INMET) situated 700 m from the experimental site and are presented in the Fig. 1.

The soil of the experimental area is a dystrophic Ferralsol (IUSS Working Group WRB 2015) with a clayey texture. Soil samples were collected from the 0–20 cm layer for chemical characterization and to establish the experiment. Soil chemical analysis showed: pH (water), 5.25; phosphorus (Mehlich-1), 14.2 mg dm^-3^; potassium, 188 mg dm^-3^; calcium, 2.5 cmolc dm^-3^; magnesium, 1.1 cmolc dm^-3^; aluminum, 0.05 cmolc dm^-3^; H + Al, 4.62 cmolc dm^-3^; base saturation, 44.5%; and organic matter, 3.1 mg dm^-3^.

Pastures of *Urochloa brizantha* cv. BRS Paiaguás (Paiaguás grass) and *Urochloa brizantha* × *Urochloa ruziziensis* cv. BRS Ipyporã (Ipyporã grass) were established in December 2016, with one hectare of each cultivar. After soil preparation with plowing, harrowing, and liming, sowing was carried out by broadcast seeding using 5 kg ha^− 1^ of pure live seeds concomitantly with phosphorus fertilization, with 80 kg ha^− 1^ of P2O5 (single superphosphate).

In February 2017, a conditioning grazing was performed with Holstein × Gyr crossbred dairy heifers (*Bos taurus taurus* L. × *Bos taurus indicus* L.) with an average body weight of 200 ± 15 kg. Subsequently, the experimental paddocks were subdivided (ten paddocks of 1.000 m² for each cultivar) for pasture management and conditioning under rotational stocking (with dry cows) throughout the year 2017. During the rainy season, the pastures were broadcast-fertilized with the equivalent of 40 kg ha^− 1^ of N per grazing cycle, using urea (46% N) as the source, immediately after the animals left the paddocks.

### Experimental treatments and statistical design

The experiment was divided into three experimental phases: phase 1, from December 04, 2017 to January 28, 2018; phase 2, from February 05 to April 03, 2018; and phase 3, from December 16, 2018 to March 01, 2019. Each experimental phase consisted of three grazing cycles (each grazing cycle corresponded to the period in which the animals grazed from paddock 1 to paddock 10). Treatments consisted of the two cultivars and the three evaluation phases. For milk yield and milk composition, a complete switchback trial was conducted with five testers cows arranged in three periods. For agronomic evaluations, a completely randomized design was used in a 2 (two cultivars under study) × 3 (experimental phases) factorial arrangement, with four paddocks evaluated for each treatment per grazing cycle. The paddock was considered as the replicate for the treatments. Dry matter intake was evaluated using a cross-over design, and was conducted in two evaluations, the first (intake 1) between February 28 and March 28, 2018, and the second (intake 2) between January 08 and February 25, 2019, with 5 replicates per treatment.

### Grazing method, growth characteristics, and morphological composition of the pasture

The grazing method used was the rotational stocking. The criterion adopted to determine the time of animal entry into the paddocks was the canopy reaching 95% light interception (LI) (Silva and Nascimento 2007). Light interception was measured weekly and was estimated as the mean of ten points in each paddock using the Accupar LP 80 device from DECAGON (USA). The post-grazing canopy residual height for each cultivar corresponded to 50% defoliation of the pre-grazing height (Sbrissia et al. 2018). To achieve the residual height, stocking adjustments were performed using the put-and-take technique so that defoliation was achieved within two days of occupation (Allen et al. 2011). Thus, when necessary, extra dry cows were placed in the paddocks to ensure the residual canopy height within the predetermined period.

Canopy height was evaluated using a centimeter-graduated ruler, with 30 random points measured per paddock, both in pre-grazing and post-grazing conditions.

Total herbage mass (HM), under pre- and post-grazing conditions, was estimated using the direct (destructive) method. For this, a 1.0 × 0.5 m (0.50 m²) metal frame was used and placed at four points representative of the average canopy height in each evaluated paddock. The material within each quadrat was cut at ground level and then taken to the laboratory for weighing and subsequent processing.

Herbage accumulation (HA) was calculated as the difference between herbage mass in the previous post-grazing and the current pre-grazing. To determine the herbage accumulation rate (HAR), accumulation values were divided by the number of rest days between each grazing cycle. Total herbage accumulation (THA) over the experimental period was calculated by summing the accumulations of all grazing cycles.

For the morphological evaluation of the herbage components, a subsample of approximately 400 g was taken from the samples collected to determine HM under pre and post-grazing conditions. This subsample was divided into leaf blade, stem (stem + sheath), and dead material fractions, which were weighed and dried in a forced-air oven set at 55 °C for 72 h, or until constant weight. Herbage mass values were converted to kg DM ha^− 1^, and morphological components were expressed as a proportion (%) of total HM.

### Determination of chemical composition

To determine the herbage chemical composition, the samples were collected above the residual height (50% of the pre-grazing height), using the simulated grazing technique. In each grazing cycle, herbage samples were collected from four paddocks per cultivar, with each paddock considered one experimental repetition. For each paddock, four 0.5 m^2^ quadrats were placed to guide the simulated grazing sampling. Samples of the energy supplement were collected weekly. After collection, herbage samples were pre-dried in a forced-air oven (55 °C; until constant weight). Subsequently, herbage and supplement samples were ground in a Wiley mill equipped with 1 mm screens and then stored in properly identified containers. Bromatological analyses were performed according to INCT-CA (Detmann et al. 2012) to determine dry matter at 105 °C, total nitrogen, ether extract, minerals, and ash contents. Cell wall components were determined according to INCT-CA methods F-001/1 (neutral detergent fiber), F-003/1 (acid detergent fiber), and F-005/1 (lignin) (Detmann et al. 2012). In vitro dry matter digestibility was also analyzed (Tilley and Terry 1963).

### Milk production and composition

To evaluate the productive performance of lactating cows, 10 Holstein × Gyr cows were used shortly after peak lactation, with mean and standard deviations for milk yield, days in milk, and body weight of 17.38 ± 1.81 kg day^− 1^ of milk, 85 ± 10 days, and 510 ± 8 kg, respectively. Cows were allocated to the treatments according to these characteristics in order to obtain two homogeneous groups.

During the experimental period, cows were supplemented with concentrate twice a day during milking, with 2 kg day^− 1^ per cow (Table 1) in the morning (07:00 h) and 1 kg day^− 1^ per cow in the afternoon (14:00 h), totaling 3 kg day^− 1^ per cow (as fed basis). Between milking, the animals remained in a resting area. Animals underwent ectoparasite control and weighing at the end of each grazing cycle between milking.

**Table 1:** Nutritional composition of the concentrate in each experimental phase

Item	Phase 1	Phase 2	Phase 3
Dry matter (DM), g kg^− 1^	953.0	951.8	951.5
In vitro dry matter digestibility, g kg^− 1^ of DM	863.7	884.1	884.0
Ether extract, g kg^− 1^ of DM	36.2	34.4	35.2
Neutral detergent fiber, g kg^− 1^ of DM	129.6	117.5	120.5
Acid detergent fiber, g kg^− 1^ of DM	48.2	43.9	46.5
Crude protein, g kg^− 1^ of DM	202.0	214.5	210.2

Individual milk yield was measured at the end of each milking and added together to obtain milk yield per cow/day, subsequently, milk yield per hectare/day was calculated. To determine milk composition, milk samples were collected individually, refrigerated, and then sent to the Milk Quality Laboratory of Embrapa Dairy Cattle, in Juiz de Fora, MG.

### Estimation of dry matter intake

Dry matter intake (DMI) was estimated in each grazing cycle from fecal dry matter output, using titanium dioxide (TiO₂) as an external marker, and from the in vitro dry matter digestibility (IVDMD) of pasture and supplement. Titanium dioxide was orally supplied at 10 g cow⁻^1^ day⁻^1^, divided into two daily doses of 5 g each, for 12 consecutive days: the first six days were used for marker stabilization and the last six days for fecal collection. Ten cows were used for DMI estimation, five per treatment, with each cow considered one experimental replicate. Fecal samples were collected twice daily from each cow during the six-day collection period and subsequently pooled by cow within each evaluation period for TiO₂ analysis. IVDMD was determined according to Tilley and Terry (1963). Fecal samples were dried in a forced-air oven at 55 °C, ground through a 1 mm screen, and analyzed for TiO₂ concentration according to INCT-CA method M-007/1 (Detmann et al. 2012). Fecal output (FO, kg cow^− 1^ day^− 1^ of DM) was calculated using Eq. 1, as follows:

1 $${\rm{FO = }}{\matrix{{\rm{daily}}\,{\rm{marker}} \hfill \cr {\rm{dose}}\left( {{\rm{g}}\,{\rm{da}}{{\rm{y}}^{{\rm{ - 1}}}}} \right) \hfill \cr} \mathord{\left/{\vphantom {\matrix{{\rm{daily}}\,{\rm{marker}} \hfill \cr {\rm{dose}}\left( {{\rm{g}}\,{\rm{da}}{{\rm{y}}^{{\rm{ - 1}}}}} \right) \hfill \cr} \matrix{{\rm{marker}}\,{\rm{concentration}} \hfill \cr {\rm{in}}\,{\rm{fecal}}\,{\rm{DM}}\left( {{\rm{g}}\,{\rm{k}}{{\rm{g}}^{{\rm{ - 1}}}}} \right) \hfill \cr} }} \right.\kern-\nulldelimiterspace} \matrix{{\rm{marker}}\,{\rm{concentration}} \hfill \cr {\rm{in}}\,{\rm{fecal}}\,{\rm{DM}}\left( {{\rm{g}}\,{\rm{k}}{{\rm{g}}^{{\rm{ - 1}}}}} \right) \hfill \cr} }$$

Daily pasture DMI was estimated using Eq. 2:

2 $$\eqalign{& DMI\left( {kg\,co{w^{ - 1}}da{y^{ - 1}}} \right) = \cr & (\left( {FO - F{O_{conc}}} \right)/\left( {1 - \left( {IVDM{D_{pasture}}/100} \right)} \right) \cr}$$

Where: FO_conc_ (kg cow^− 1^ day^− 1^) = fecal output attributable to concentrate intake (FO_conc_ = concentrate DM intake, kg cow^− 1^ day^− 1^ × (1 – (IVDMD_conc_/100)); IVDMD_conc_ = IVDMD of the concentrate; IVDMD_pasture_ = IVDMD of the pasture.

### Statistical analyses

The assumptions of normal probability distribution and homogeneity of variances required for the statistical models were verified using the residuals obtained from the fitted models, by means of the Shapiro–Wilk and Bartlett tests, respectively (Kaps and Lamberson 2009).

Analyses of variance (ANOVA) for the agronomic variables were performed according to the following model:

$$\:{Y}_{ijk}=\hspace{0.17em}+\hspace{0.17em}{C}_{i}+{}_{ik}+{F}_{j}+{(F/C)}_{ij}+{}_{ijk}$$

Where: *Y*_*ijk*_ = observation of cultivar *i*, phase *j*, and replicate *k*; *µ* = overall mean; *C*_*i*_ = fixed effect of cultivar *i*; *θ*_*ik*_ = random error associated with the observation of cultivar *i* and replicate *k*, with *θ*_*ik*_ ~ N(*µ*,* σ*^*2*^); *Fj* = fixed effect of phase *j*; (*F/C*)_*ij*_ = interaction effect between cultivar *i* and phase *j*; *α*_*ijk*_ = random error associated with the subplot of cultivar *i* in phase *j* of replicate *k*, with *α*_*ijk*_ ~ N(*µ*,* σ*^*2*^).

Analyses of variance (ANOVA) for DMI were performed according to the following model:

$$\:{Y}_{ijk}=\mu\:\hspace{0.17em}+\hspace{0.17em}{P}_{j}+{A}_{k}+{T}_{i}+{}_{ijk}$$

Where: Y_*ijk*_ = observation in adjusted treatment *i*, in period *j* of animal *k*; *µ* = overall mean effect; *P*_*j*_ = effect of period *j*; *A*_*k*_ = effect of animal *k*; *T*_*i*_ = effect of treatment *i*; *ε*_*ijk*_ = random error of treatment *i* in period *j* of animal *k*, with *ε*_*ijk*_ ~ N(*µ*,* σ*^*2*^).

For milk yield variables, analyses of variance (ANOVA) were performed according to the following model:

$$\:{Y}_{ijk}=\mu\:\hspace{0.17em}+\hspace{0.17em}{P}_{j}+\:{T}_{i}+{}_{ijk}$$

Where: *Y*_*ijk*_ = observation in adjusted treatment *i*, in period *j* of replicate *k*; *µ* = overall mean effect; *P*_*j*_ = effect of period *j*; *T*_*i*_ = effect of adjusted treatment *i*; *ε*_*ijk*_ = random error of adjusted treatment *i* in period *j* of replicate *k*, with *ε*_*ijk*_ ~ N(*µ*,* σ*^*2*^).

To compare the means of experimental groups, orthogonal contrasts were used by the Fisher’s analysis, with an error rate lower than 5% as the criterion for statistical significance. For unfolding interactions and comparing phases, Tukey’s test was used. All analyses were performed using R Development Core Team (2019) software.

## Results

### Agronomic characteristics and morphological composition of the pasture

No significant difference (*P* > 0.05) was observed between the rest periods of the cultivars in any of the experimental phases; the observed mean was 18 days. There was no cultivar × phase interaction effect (*P* > 0.05) for the variables canopy height, morphological components under pre and post-grazing conditions, leaf/stem ratio, herbage accumulation, herbage accumulation rate and volumetric density (Table 2).

**Table 2:** Canopy height, herbage mass (HM), morphological components at pre and post-grazing, herbage accumulation (HA), herbage accumulation rate (HAR), and volumetric density (VD) of two cultivars (C) – BRS Ipyporã and BRS Paiaguás – and three experimental phases (P)

Variables	BRS Ipyporã			BRS Paiaguás			SEM	*P*-value		
	Phase 1	Phase 2	Phase 3	Phase 1	Phase 2	Phase 3		C	*P*	C*P
Pre-grazing										
Height (cm)	41.55	43.35	39.98	67.38	65.95	57.13	2.03	< 0.0001	0.0047	0.1050
HM (Kg DM ha^− 1^)	5153.19	5917.09	5059.4	8104.99	7075.39	6232.35	537.71	< 0.0001	0.0156	0.0184
L/S	1.54	1.41	1.67	0.9	0.96	1.21	0.06	< 0.0001	0.0009	0.4569
Leaf (%)	49.78	50.21	50.87	38.19	39.17	44.48	1.92	< 0.0001	0.1330	0.3371
Stem (%)	33.77	36	30.96	42.98	41.02	37.11	1.35	< 0.0001	0.0017	0.2867
Dead material (%)	16.44	13.78	18.16	18.43	18.91	18.2	2.14	0.1773	0.6929	0.4915
Post-grazing										
Height (cm)	22.22	20.87	20.48	35.75	33.19	30.44	0.92	< 0.0001	0.0014	0.1538
HM (Kg DM ha^− 1^)	2747.05	2236.62	1992.65	3781.2	3228.99	2767.01	176.4	< 0.0001	0.0005	0.7834
Leaf (%)	19.76	22.18	18.18	14.43	13.92	11.61	1.24	< 0.0001	0.0412	0.5036
Stem (%)	46.28	43.85	44.4	47.83	45.36	37.72	1.76	0.4041	0.0046	0.0319
Dead material (%)	33.95	33.96	37.4	37.73	40.71	50.66	2.18	< 0.0001	0.0008	0.0939
HA (kg DM ha^− 1^ phase^− 1^)	4714.95	7180.35	6453.99	8645.07	8982.35	9207.19	426.21	0.0018	0.0042	0.0543
HAR (kg DM ha^− 1^ day^− 1^)	78.58	119.67	85.81	144.08	157.69	118.77	9.76	0.0009	0.0016	0.2121
VD (kg DM cm ha^− 1^)	117.07	141.14	125.29	108.39	103.88	112.36	8.1	0.0029	0.5209	0.1680

L/S = Leaf/Stem ratio; SEM = Standard error of the mean

The canopy height differed (*P* < 0.05) between cultivars under pre and post-grazing conditions (Table 2). Paiaguás grass showed a 1.52 times greater management height than Ipyporã grass at pre-grazing and 1.57 times at post-grazing. In addition, pre and post-grazing herbage mass differed significantly between cultivars (*P* < 0.05). Under pre-grazing conditions, HM of BRS Paiaguás grass exceeded that of Ipyporã grass by 1,761 kg DM ha^− 1^. Under post-grazing conditions, HM was 933.41 kg DM ha^− 1^ higher in Paiaguás grass. The leaf/stem ratio (L/S) was 33.76% higher in Ipyporã grass (1.54 vs. 1.02; *P* < 0.05).

The leaf proportion was also higher in Ipyporã than in Paiaguás grass (*P* < 0.05), both at pre-grazing (50.3 vs. 40.6%, respectively) and post-grazing (20 vs. 13.3%, respectively; Table 2). At pre-grazing, stem proportion was 16.81% higher (*P* < 0.05) in Paiaguás grass, whereas at post-grazing no difference was observed between cultivars. The proportion of dead herbage mass did not differ between the cultivars (*P* = 0.17), but it was higher (*P* < 0.05) in the residue of Paiaguás grass.

Herbage accumulation (HA) and herbage accumulation rate (HAR) differed between the evaluated cultivars (*P* < 0.05; Table 2). Both variables were higher in Paiaguás grass: HA was 31.61% and HAR was 32.43% higher than in Ipyporã grass. For volumetric density (VD), there was a significant difference between cultivars (*P* < 0.05). VD was 15% higher in Ipyporã grass compared with Paiaguás.

Among the phases, a significant difference (*P* < 0.05) was observed for HM, canopy height, L/S, and stem percentage at pre-grazing, being lower in the phase three. Under post-grazing conditions, canopy height, HM, and leaf and stem percentages were higher in phase 1 (*P* < 0.05). Herbage accumulation and HAR also differed among the experimental phases (*P* < 0.05), being higher in phase two (8,081.35 kg DM ha^− 1^ phase^− 1^ and 138.68 kg DM ha^− 1^ day^− 1^, respectively) (Table 3). Volumetric density did not differ among phases (*P* = 0.52), and the observed mean was 117.96 ± 7.71 kg DM cm ha^− 1^.

**Table 3:** Canopy height, herbage mass (HM), leaf/stem ratio (L/S), morphological components, herbage accumulation (HA), herbage accumulation rate (HAR), and volumetric density (VD) at pre and post-grazing and three experimental phases

Variables	Phase			SEM	*P*-value
	1	2	3		
	Pre-grazing				
Height (cm)	54.46 a	54.65 a	48.56 b	1.43	0.0047
HM (Kg DM ha^− 1^)	6629.09 a	6496.22 a	5645.88 b	380.22	0.0156
L/S	1.22 b	1.18 b	1.44 a	0.56	0.0045
Leaf (%)	43.98 a	44.69 a	47.68 a	1.35	0.1330
Stem (%)	38.38 a	38.51 a	34.04 b	0.96	0.0017
Dead material (%)	17.43 a	16.34 a	18.18	1.15	0.6929
	Post-grazing				
Height (cm)	28.98 a	27.03 ab	25.46 b	0.65	0.0013
HM (Kg DM ha^− 1^)	3262.45 a	2732.80 b	2379.83 b	150.63	0.0005
Leaf (%)	17.09 ab	18.05 a	14.89 b	0.88	0.0412
Stem (%)	47.05 a	44.61 ab	41.06 b	1.24	0.0046
Dead material (%)	35.84 b	37.33 b	44.03 a	1.54	0.0008
	Accumulation				
HA (kg DM ha^− 1^ Phase^− 1^)	6680.01 b	8081.35 a	7830.59 a	301.58	0.0042
HAR (kg DM.ha^− 1^ dia^− 1^)	111.33 b	138.68 a	102.29 b	6.90	0.0016
	Volumetric density				
VD (kg DM cm ha^− 1^)	112.57 a	122.51 a	118.82 a	7.71	0.5209

Lowercase letters compare phases by Tukey’s test (*P* < 0.05). SEM = Standard error of the mean

**Fig. 2:**
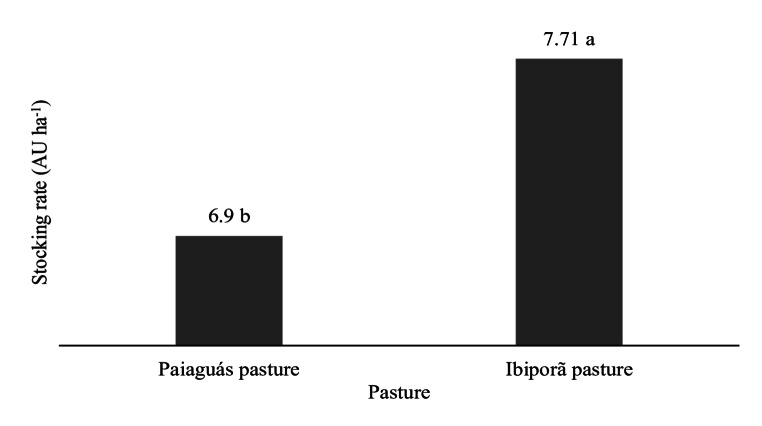
Stocking rate of Paiaguás and Ipyporã grass pastures, expressed as the overall mean across the three experimental phases. Lowercase letters compare cultivars by Fisher’s test (*P* < 0.05); AU = animal unit (450 kg); ha = hectare; SEM = 0.23; *P* = 0.03

The stocking rate (SR) (AU ha^− 1^) differed between cultivars (*P* < 0.05), being 11% higher in Paiaguás grass (Fig. 2).

### Herbage chemical composition

For fiber composition and IVDMD, a significant interaction (*P* < 0.05) was observed between the factors under study (Table 4). Neutral detergent fiber (NDF) and acid detergent fiber (ADF) were higher in Paiaguás grass in all evaluated phases. Regarding comparisons among phases, Paiaguás grass showed higher NDF and ADF in phase three, whereas Ipyporã grass showed higher values in phase two. The lignin content was higher in Paiaguás grass than in Ipyporã grass. When comparing phases, there was a difference only for Ipyporã grass, for which the lowest lignin content was observed in phase three. IVDMD differed between cultivars in experimental phase three, being higher in Ipyporã grass. Among phases, for both cultivars the highest digestibility occurred in phase three.

**Table 4:** Fiber composition and in vitro dry matter digestibility (IVDMD) of the cultivars BRS Paiaguás and BRS Ipyporã across three experimental phases

Cultivar	Phase			SEM	P-value
	1	2	3		
	NDF (g kg^−1^)				
BRS Ipyporã	566.73 bB	590.55 bA	566.03 bB	7.01	0.0008
BRS Paiaguás	626.85 aAB	614.85 aB	644.28 aA		
	ADF (g kg^−1^)				
BRS Ipyporã	260.57 bB	283.35 bA	262.93 bB	3.76	0.0001
BRS Paiaguás	314.81 aAB	310.68 aB	325.69 aA		
	Lignin (g kg^−1^)				
BRS Ipyporã	22.88 bA	23.29 bA	19.7 bB	0.87	0.0478
BRS Paiaguás	30.59 aA	28.05 aA	28.56 aA		
	IVDMD (g kg^−1^)				
BRS Ipyporã	700.80 aB	710.50 aB	830.77 bA	5.64	0.0500
BRS Paiaguás	700.00 aB	714.94 aB	808.56 aA		

Lowercase letters compare cultivars and uppercase letters compare phases by Tukey’s test (P < 0.05). NDF = neutral detergent fiber; ADF = acid detergent fiber; SEM = standard error of the mean

**Fig. 3:**
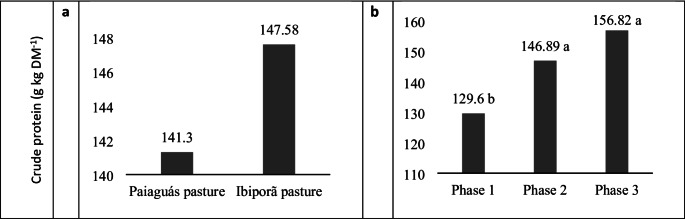
Crude protein content (**a**) of BRS Paiaguás and BRS Ipyporã across three experimental phases (**b**). Lowercase letters compare phases by Tukey’s test (*P* < 0.05); a: SEM-cultivar = 2.89; P-cultivar = 0.416; b: SEM-phase = 3.58; P-Phase < 0.0001

Crude protein content did not differ between cultivars (*P* > 0.05) with a mean value of 144.44 g kg^− 1^. Among phases, the lowest crude protein content was observed in phase 1, with an observed mean of 129.60 g kg^− 1^ (Fig. 3).

### Dry matter intake

The herbage DMI differed between cultivars only in the first intake evaluation (*P* < 0.05), when it was higher for Ipyporã grass (Table 5). In the second intake evaluation, there was no difference between cultivars, and the observed mean was 11.68 ± 0.42 kg cow^− 1^ day^− 1^.

**Table 5:** Herbage dry matter intake (DMI) of Holstein × Gyr cows managed under grazing on BRS Ipyporã and BRS Paiaguás pasture

Variable	Intake 1		SEM	*P*-value	Intake 2		SEM	*P*-value
	BRS Ipyporã	BRS Paiaguás			BRS Ipyporã	BRS Paiaguás		
DMI (kg cow^− 1^ day^− 1^)	11.11 a	10.02 b	0.22	0.0125	12.05 a	11.31 a	0.42	0.2541
DMI (% of BM)	2.21 a	1.97 b	0.08	0.0122	2.43 a	2.28 a	0.13	0.3251

Lowercase letters compare cultivars by Fisher’s test (*P* < 0.05); SEM = standard error of the mean; BW = body weight

### Milk production and composition

No significant difference was observed in individual daily milk production (L cow^− 1^ day^− 1^), daily milk production per area (L ha^− 1^ day^− 1^) nor in the analyzed milk composition variables (fat, protein, lactose, and total solids) (Table 5).

**Table 6:** Individual Milk yield, milk yield per hectare and milk composition of Holstein × Gyr cows managed under grazing on BRS Ipyporã and BRS Paiaguás pasture across three experimental phases

Phase	Cultivar			
	BRS Ipyporã	BRS Paiaguás	SEM	*P*-value
	Individual milk yield (L cow^− 1^ day^− 1^)			
1	15.49	15.45	0.69	0.94
2	13.93	13.84	0.46	0.84
3	13.71	14.00	0.72	0.66
	Milk yield (L ha^− 1^ day^− 1^)			
1	106.33	109.66	4.49	0.43
2	102.94	108.31	3.79	0.13
3	109.09	109.56	5.68	0.92
	Fat (g kg^− 1^)			
1	37.2	32.7	2.2	0.18
2	31.9	31.9	0.8	0.96
3	31.0	31.7	0.5	0.40
	Protein (g kg^− 1^)			
1	28.7	27.9	0.7	0.45
2	29.2	25.0	3.0	0.33
3	33.8	29.5	1.8	0.11
	Lactose (g kg^− 1^)			
1	46.7	46.8	0.74	0.98
2	47.0	46.9	0.78	0.93
3	45.3	46.6	0.78	0.23
	Total solids (g kg^− 1^)			
1	122.0	126.0	3.7	0.37
2	117.0	113.0	2.8	0.33
3	120.0	118.0	1.9	0.47

SEM = Standard error of the mean

## Discussion

The goals for animal entry and exit from paddocks chosen in the study (95% LI for animal entry and a 50% reduction in height) aimed at the most efficient use of the herbage and they were achieved (Da Silva and Nascimento Jr 2007; Sbrissia et al. 2018). The management height of the cultivars was higher than those observed in other studies. Echeverria et al. (2016) found a mean height of 30 cm when Ipyporã grass was managed at 95% LI under intermittent grazing, a value similar to that observed by Paraiso et al. (2019) of 29 cm under continuous stocking. In a study with Paiaguás grass, the height observed by Gobbi et al. (2018) was 34 cm in plots managed at 95% LI. Under continuous stocking management, Euclides et al. (2016) proposed a management height of 30 cm.

Among the experimental phases, the difference in heights is associated with management adjustment throughout the experiment (Table 3). The greater canopy height found in phase one can be explained by the pre-experimental pasture conditions, which presented high HM, stem, and dead material, mainly for Paiaguás grass (Table 2). These results corroborate the findings of Euclides et al. (2019), who observed that a greater management height of Marandu grass (*Urochloa brizantha* cv. Marandu) resulted in a higher stem percentage and, consequently, a lower L/S ratio. As the experimental phases progressed, heights were adjusted, and in phase three the pre-grazing height was 11% lower than those observed in phases 1 and 2 (Table 3).

Under Brazilian semiarid conditions, Rodrigues et al. (2021) reported that a canopy height of 46.3 cm resulted in 78.7% LI during the establishment of Paiaguás grass. Establishing pastures have greater spacing between clumps and, consequently, insufficient canopy density to cover the soil. In this situation, canopies do not have sufficient density to determine a management height associated with the recommended LI. As grazing occurs, the tillers that emerge tend to be smaller than those that were grazed (Gastal and Lemaire 2015) but the tiller density increase (Hodgson 1990). This explains the greater heights in the first phase, decreasing up to the third phase for both *Urochloa* cultivars (Table 2).

A 50% reduction in canopy height through grazing is considered moderate, ensuring high herbage intake rates with great nutritive value (Fonseca et al. 2012; Mezzalira et al. 2014; Sbrissia et al. 2018). Thus, DMI is expected to be higher (Zanine et al. 2018) and animals consume more leaf, since 90% of stem mass is concentrated in the lower half of the herbage canopy (Zanini et al. 2012).

One of the factors that most affects the profitability of pasture-based dairy systems is the productivity (Hanrahan et al. 2018). Thus, the greater the quantity and the quality of available herbage, the more promising pasture-based milk production systems tend to be (Elgersma et al. 2015).

The HM observed in the pastures was within the range reported in the scientific literature, varying from 3,095 to 6,110 kg DM ha^− 1^ for Ipyporã grass (Echeverria et al. 2016; Euclides et al. 2018; Paraiso et al. 2019) and from 2,605 to 8,115 kg DM ha^− 1^ for Paiaguás grass (Euclides et al. 2016; Germano et al. 2018; Gobbi et al. 2018). Ipyporã grass showed lower HM compared with Paiaguás grass (Table 2), which may be related with the lower productivity of the Ipyporã grass progenitor, *Urochloa ruziziensis*, compared with other *Urochloa* species (Euclides et al. 2018; Paraiso et al. 2019). This resulted in a lower SR than that of Paiaguás grass pasture (Fig. 2).

However, Ipyporã grass showed a higher leaf percentage, a lower stem percentage and, consequently, a higher L/S ratio (Table 3), highlighting it compared to other *Urochloa* spp. Cultivars (Valle et al. 2017). Gurgel et al. (2022) also found a greater leaf blade mass and L/S ratio in Ipyporã grass during establishment in the Cerrado biome, concluding that the stem elongation rate of Paiaguás grass was higher than that of Ipyporã grass (1.2 and 0.52, respectively).

The greater fiber fraction and the lower digestibility observed in Paiaguás grass are explained by its higher stem percentage (Table 4). The stem is the structural component of the plant, in which a greater amount of fibrous tissues is concentrated (Van Soest 1994). Crude protein content, however, did not differ between cultivars (Fig. 3).

The management of canopy height directly affects the morphological components of the herbage canopy (Silva Neto et al. 2020; Mezzalira et al. 2014). An increase in canopy height results in competition for light among plants, promoting stem elongation in order to take advantage of higher light incidence (Moura et al. 2017).

During the phase three, the management had already been stabilized, therefore, a lower pre-grazing height was maintained (Table 3). A consequence of the management adjustment was the higher L/S ratio and the lower stem percentage in this phase (Table 3). Similar behavior was observed by Echeverria et al. (2016), in which a greater management height resulted in a decrease in the L/S ratio in Marandu grass. The reduction in pre-grazing height influenced the chemical composition of the cultivars (Euclides et al. 2019), showing higher IVDMD (Table 4) and crude protein (Fig. 3) values compared with experimental phases one and two.

The potential of the herbage production for each grass is genetically determined, but it depends on environmental conditions (water, light, temperature, and nutrient availability). Grazing management is also fundamental for the productive potential to be achieved (Sbrissia et al. 2018). Herbage accumulation after defoliation results from the flow of new tissues (Hodgson 1990), which occurs both in defoliated but not decapitated tillers and in new tillers.

The high HAR observed in the study (between 94.69 and 140.15 kg DM ha^-1^ day^-1^) reflects the favorable climatic conditions during the experimental period (Fig. 1), as well as the level of fertilization applied and the great response of the cultivars to fertilization. In intensive production systems, rapid regrowth, represented by a short interval between grazing events, allows higher herbage quality and a reduction in the number of paddocks in the rotational stocking method (Gomide and Paciullo 2014). Therefore, it is necessary to highlight the rapid attainment of grazing readiness of the cultivars (95% LI) during the rainy season, resulting in a mean rest period of 18 days.

The lower herbage accumulation (HA) observed in phase one may be associated with the greater management height and lower volumetric density (VD) during this phase (Table 3). Pastures managed at greater heights tend to undergo stem elongation, reducing the canopy density and, consequently, the herbage accumulation (Euclides et al. 2019; Moura et al. 2017). This explains the higher HA and L/S observed in the phases in which management was already established (phases two and three). During phase three, an extended dry spell occurred, increasing the days required for the herbage to reach its optimal grazing point (95% LI), and significantly reducing the HAR (Table 3) (Beloni et al. 2018).

The higher volumetric density (kg DM cm^-^¹ ha^-^¹) observed in Ipyporã grass (Table 2) can be explained by its lower average height, since this cultivar was managed at a height 35% lower than that of Paiaguás grass, while its herbage mass was only 25% lower (Table 2).

Volumetric density and the HM are important variables that directly affects the accessibility of herbage to be consumed by animals (Hodgson 1990). The higher VD of Ipyporã grass, combined with a higher leaf percentage, higher L/S ratio, and lower fibrous fraction, explains the greater DMI observed in the first intake evaluation (intake 1) (Table 5) compensating for its lower HM compared to Paiaguás.

Stocking rate adjustment was performed based on the HM produced, which explains the higher SR observed for Paiaguás grass (Fig. 2), due to its greater dry matter (DM) yield. A similar relationship was reported by Moura et al. (2017), who found a higher SR in treatments with Marandu grass managed at 95% light interception compared with treatments managed under fixed rest periods.

The higher SR at Paiaguás pasture was not enough to impact daily milk productivity (L ha^-1^ day^-1^). Morphological and chemical composition indicated a better structural and nutritional conditions at pre-grazing in Ipyporã grass pastures (Tables 2 and 4), directly affecting the DMI (Fonseca et al. 2013). Under grazing conditions, DMI is directly influenced by non-nutritional factors, which vary according to the structure of the herbage canopy (Da Silva et al. 2012). Grasses with a higher leaf proportion, greater VD, and lower fibrous fraction are preferentially more consumed (Mezzalira et al. 2014; Moura et al. 2017).

Adjustments in herbage management can result in improvements in bromatological composition and in herbage intake (Anjos et al. 2016; Pereira et al. 2015). These arguments are supported by the values observed in the present study, since adjustments in the management height of the cultivars during the third experimental phase (intake 2) resulted in a higher L/S ratio, greater in vitro dry matter digestibility (IVDMD) (Table 4) and equalized DMI between cultivars.

The values observed for DMI were close to those reported for other tropical grasses, with an average of 13 kg cow^-^¹ day^-^¹ in Tanzania grass pasture (*Megathyrsus maximus* cv. Tanzania), 11.3 kg cow^-1^ day^-1^ in stargrass cv. Africana (*Cynodon nlemfuensis*) and 11.7 kg cow^-1^ day^-1^ in Marandu grass. Fukumoto et al. (2010) found a DMI of 10.1 and 12.3 kg cow^-^¹ day^-^¹ in pastures of elephant grass cv. Cameroon (*Cenchrus purpureus*) managed under maximum light interception and 95% light interception, respectively, showing the detrimental effect of stem accumulation on DMI by grazing animals.

The milk yield observed in this study (Table 5) is within the range reported in the literature for cows grazing tropical grass pastures. In a study with elephant grass cv. Cameroon, milk production values ranged from 15.7 to 18.1 L day^-1^ (Congio et al. 2018). Demski et al. (2019) reported milk yields between 17.34 and 13.73 L cow^-1^ day^-1^ for the Mulato II hybrid (*U. brizantha* × *U. ruziziensis* × *U. decumbens*) and between 17.24 and 11.96 L cow^-^¹ day^-^¹ for Marandu grass. When evaluating Marandu grass at 30 days of regrowth and 95% light interception, milk production ranged from 100.3 to 139.7 L ha^-1^ day^-1^ (Moura et al. 2017). However, it is worth noting that this study used cows with higher production potential, as well as a higher level of concentrate supplementation (6 kg cow^-^¹ day^-^¹). To date, there are no data in the literature regarding milk production in pastures of BRS Ipyporã and BRS Paiaguás.

Milk solids content (Table 5) was similar to the values reported by Demski et al. (2019), who evaluated grazing on Marandu grass and Mulato II grass and observed mean values of 39.15 g kg^-1^ for fat, 34.75 g kg^-^¹ for protein, 42.7 g kg^-1^ for lactose, and 126.45 g kg^-1^ for total solids.

The similarity between the values observed in this study and those reported in the literature indicates the potential of Paiaguás and Ipyporã grasses to be used in pasture-based dairy production systems. These values are also close to those observed in studies with elephant grass by Voltolini et al. (2010), as well as in studies with Tanzania grass, Marandu grass, and star grass (Porto et al. 2009).

The lack of differences in milk yield and milk composition between the cultivars can be explained by the fact that they belong to the same genus, were managed under the same conditions (95% light interception and 50% post-grazing height reduction), which allowed greater leaf selection by the cows, and received the same amount of concentrate supplementation (3 kg cow^-1^ day^-1^).

## Conclusion

The cultivars evaluated show potential for use in pasture-based dairy production systems when managed at 95% light interception (41 cm pre-grazing height for cv. BRS Ipyporã and 63 cm for cv. BRS Paiaguás) with a 50% post-grazing height reduction.

The BRS Ipyporã cultivar showed a higher leaf proportion and a greater leaf-to-stem ratio, resulting in herbage with lower fiber and lignin content. However, BRS Paiaguás produced a greater herbage mass, resulting in a higher stocking rate.

The average milk productivity of BRS Paiaguás and BRS Ipyporã pastures under rotational grazing during the rainy season is approximately 107 L ha^− 1^ day^− 1^.

## Data Availability

Data sets generated during the current study are available from the corresponding author on reasonable request.
